# LC-PROM: Validation of a patient reported outcomes measure for liver cirrhosis patients

**DOI:** 10.1186/s12955-016-0482-y

**Published:** 2016-05-10

**Authors:** Ying Zhang, Yuanyuan Yang, Jing Lv, Yanbo Zhang

**Affiliations:** Department of Health Statistics, School of Public Health, Shanxi Medical University, 56 South XinJian Road, Taiyuan, Shanxi Province 030001 People’s Republic of China

**Keywords:** Liver cirrhosis, Patient-reported outcome (PRO), Item selection, Item Response Theory (IRT), Confirmatory factor analysis (CFA), Reliability, Validity

## Abstract

**Background:**

The aim of the study is to develop a specific patient-reported scale of liver cirrhosis according to the Patient Reported Outcome guidelines of the Food and Drug Administration (FDA), and to examine its capacity to fill gaps in this field.

**Methods:**

A conceptual framework was developed and a preliminary item pool developed through literature review and interviews of 10 patients with liver cirrhosis. With the preliminary items, we performed a pilot survey that included a cognitive test with patients and interviews with experts; the focus was on content and language of the scale. In the item selection stage, seven statistical methods including discrete trends method, discrimination analysis, exploratory factor analysis, Cronbach’s α coefficient, correlation coefficient, test-retest reliability, Item-Response Theory were applied to survey data from 200 subjects (150 liver cirrhosis patients and 50 controls). This produced the preliminary Liver Cirrhosis Patient-reported Outcome Measure (LC-PROM). In the next stage, we conducted the survey with 620 subjects (500 patients and 120 controls) to validate reliability, validity and acceptability of this scale.

**Results:**

The 55 items and 13 dimensions addressed four domains: physical, psychological, social, and therapeutic. Cronbach’s α coefficients were 0.921 for the total scale; the confirmatory factor analysis, t-tests and ANOVA supported scale validity; the model fit index as Root Mean Square Error of Approximation (RMSEA), Root Mean Square Residual (RMR), Normed Fit Index (NFI), Non-Normed Fit Index (NNFI), Comparative Fit Index (CFI) and Incremental Fit Index (IFI) met the criterion generally. The acceptance ratio and response rate indicated good feasibility.

**Conclusions:**

This study developed an accurate and stable patient-reported outcome scale of liver cirrhosis, which is able to evaluate clinical effects effectively, is helpful to patients in recognizing their health condition, and contributes to clinical decision making both for patients and physicians. Additionally, the LC-PROM can perform as an ultimate assessment of medical and health care effects and can inform clinical trials of new drugs for liver cirrhosis.

## Background

Liver cirrhosis (LC) is a potential consequence of the progression of any of various kinds of liver disease, and the high incidence of hepatitis will lead to a large number of patients suffering from liver cirrhosis. LC is characterized by fatigue, digestive disorders, bleeding and anemia, endocrine disorder, hypoproteinemia, portal hypertension and other serious symptoms that cause great pain to patients physically, impacting their daily social life. As an irreversible, chronic, progressive disease. LC can not be cured completely at the present stage. Particularly for weak patients, the common treatments used in the clinical can cause secondary damage in addition to harm caused by the disease itself.

At present, patients’ health status and treatment effects are evaluated by hepatic function test and serological markers, or reflected by hospital stays and symptom improvement over time. However, with the continued development of a biopsychosocial medical model the use of scales to assess patients’ fitness has been widely accepted and applied internationally; that is, patients’ personally reported data, dubbed patient-reported outcome (PRO), are used to measure clinical results. One of the arguments for using questionnaires to ask patients to judge their own health-related quality of life (HRQoL) is that it has been shown that physicians are generally unable to make accurate judgments of patients’ HRQoL. Physicians’ judgments not only deviate from those of patients, they also differ among one another. This latter variability makes it particularly difficult to obtain ‘objective’ judgments of HRQoL [1].

The PRO Harmonization Group, which consists of the Food and Drug Administration (FDA), International Society For Pharmacoeconomics and Outcomes Research (ISPOR), the European Regulatory Issues on Quality of Life Assessment Group (ERIQA), and the International Society for Quality of Life Studies (ISQOL), proposes that evaluation of clinical curative effects should contain data from physicians’ reports, physiological measures, caregivers’ reports, and PROs, which come solely from the patient. In the course of a disease, there are some symptoms that can only be experienced by patients; i.e., these symptoms cannot be reflected by physical measures. In this case, the normal reference values of medicine do not equal true health; additionally, physician report data are always processed through the subjective consciousness and may only include contents related to the physician’s concerns. What’s more, this report is limited by physicians’ knowledge and experience. Therefore, PROs play an important role in clinical practice, and this method is now generally accepted by experts and patients alike. Since the publication of the draft guide for new drug development and curative effect evaluation in February 2006 [2], PROs are becoming more important in assessment of treatment outcome and in new drug registration.

A PRO instrument specific to LC could provide several benefits: it could help improve the evidence base through research assessing effectiveness of LC therapies; facilitate clinician-patient communication and shared decision making; help prioritize patient problems and preferences; monitor changes or outcomes of treatment; measure the performance of healthcare providers and services; and be incorporated in clinical audits [3–5].

In short, the aim of this study is to develop such a PRO scale that meets the following criteria: (I) specific to liver cirrhosis; (II) addresses all physical symptoms, psychological feelings, daily activities, and therapeutic status related to LC; (III) comprises items that are founded on the patients’ own perspective; (IV) has good internal consistency, a reasonable theoretical framework and can distinguish different severities of the disease; and (V) is of appropriate length and has strong feasibility.

## Methods

The Medical Ethics Committee of Shanxi Medical University provided ethics approval, and all participants signed informed consent to participate.

### Step 1 item generation

#### Literature review

We conducted literature searches on databases and network resources for PRO instruments. From the searches, we formed the conceptual framework of the new instrument, called the Liver Cirrhosis Patient Reported Outcome Measure (LC-PROM).

### Patient interviews

We conducted semi-structured interviews with ten liver cirrhosis patients (five males and five females; average age 53 years). In the interview, patients were encouraged to talk about their main disease symptoms, physical feelings and symptoms that they most desired to improve, psychological conditions after diagnosis and participation in social activities since diagnosis, adherence to therapy and satisfaction with their status. In addition, patients could speak freely on other relevant topics. Throughout the process, researchers wrote down the interviewees’ original words as far as possible, and audio recordings were made. After the interview, all information was sorted and then an initial list of items was developed.

### Cognitive debriefing and discussion with experts

Another ten patients (five males and five females, average age 52 years) were selected to undertake cognitive debriefing. These patients were asked to flag items that were ambiguously worded or difficult to understand, and to suggest items that needed to be added or deleted.

Seven experienced experts including three chief physicians of gastroenterology, one infectious diseases physician, one psychologist, one sociologist, and one ethics expert were invited to discuss whether the initial structural framework was reasonable and whether the items covered all areas of disease evaluation. The correlation of items with their respective dimensions and linguistic issues were considered. We modified the item pool according to the experts’ advice, and the preliminary scales were formed.

### Step 2 item selection

#### Sampling survey

Two hundred subjects were sampled from inpatients of eight different hospitals and communities in Shanxi Province. There were 150 LC patients and 50 health controls.

Patients who were diagnosed with definite LC, who were between 18 and 72 years old, and who were fully able and willing to participate in this study as volunteers were included.

Patients were excluded if they had an uncertain diagnosis, suffered mental illness or disorders of consciousness, were unable to understand questions because of dysgnosia, or were unable to complete the test.

Health controls were healthy volunteers from communities who were not diagnosed with any diseases by physicians and had an age distribution similar to that of LC patients. Health controls also provided informed consent and got some rewards.

The survey was administered by trained investigators. Before beginning, subjects were informed of the survey objective and signed the informed consent form. Next, the participants independently completed the preliminary scale. During the survey, investigators were present to respond to questions. If participants were elderly or had a low education level, investigators read the items to them and wrote down their answers. After the survey, any incomplete scales were filled in by the subjects under the guidance of the investigators.

### Scale scoring

Scores were calculated using a five-point Likert scale to reflect frequency of occurrence over the past 2 weeks of the issue presented in each item. The responses were 0 = never, 1 = occasionally, 2 = about half of the time, 3 = often, and 4 = almost every day. The positively-toned items were scored as the original score plus one, and the negatively-toned items were scored as 5 minus the original score. Thus every item score ranged from 1 to 5, with higher scores denoting more positive outcomes.

### Statistical methods for item selection

Item reduction was based on both Classical Test Theory (CTT) and Item Response Theory (IRT). This study employed six methods of CTT followed by IRT.

### Discrete trend

A low discrete degree means subjects were inclined to select the same answer; that is, the items had a low capacity to test for differences. In general, scores obey a normal distribution, so the standard deviation for every item was calculated. The items with a low standard deviation (<1.0) were deleted.

### Discrimination analysis

Items that do not reflect different characteristics of subjects should not remain in the scale. We compared every item score with two independent-sample t-tests (α = 0.05), and the items that were not statistically different were deleted.

### Exploratory factor analysis (EFA)

Taking the small sample size into consideration, we did EFA in each domain (physical, psychological, social, and therapeutic) separately, then rotated the solution. According to the eigenvalue and the variance contribution ratio, the number of factors was determined. Items with low factor loading (<0.4) and cross-loading on two or more dimensions were removed.

### Cronbach’s α if item deleted (CAID)

Internal consistency was evaluated with CAID and the Corrected Item Total Correlation (CITC). If the α coefficient increased greatly when an item was deleted, the item was reducing the internal consistency of its own dimension. CITC < 0.4 indicates an item poorly contributing to the construct of the scale; therefore such items were deleted.

### Correlation coefficient

The representativeness of an item was measured by its correlation coefficient with the dimension to which the item belonged. When the value was less than 0.6, the item was not retained.

### Retest reliability

This method considered item stability. Thirty subjects were selected from the sample to take a retest 2 weeks after the first test. Among these, 20 cases whose data were error-free in both tests were used to calculate retest correlation coefficient. The criterion for reliability was 0.7.

### Item response theory (IRT)

IRT is part of modern measurement theory and was put forward to overcome defects of CTT [6]. It is also called latent trait theory, and has advantages for item selection and test construction. It claims that there is a functional relationship between subjects’ abilities and their responses to an item. How to define this relationship is the basic idea and the starting point. In brief, IRT can be viewed as a probabilistic method for discussing the relationship between subjects’ potential traits and their responses to items.

If *θ* represents a subject’s ability, *P(θ)* is the probability of the subject’s responding to an item correctly; their functional relationship can be reflected by a curve called the item characteristic curve (ICC). Two important parameters on the curve are used in this study: *a* reflects discriminant degree and *b* shows item difficulty. On the ICC whose *X,Y* axes are *θ* and *P(θ), b* is the value of *θ* corresponding to *P(θ)* = 0.50; this value ranges from −3 to 3. *a* is the function of the tangent line’s slope at point *b*; its value ranges between 0.3 and 2, with larger values representing higher degrees of discrimination.

Because the five-point Likert scale was being used, a Graded Response model was constructed, which is appropriate for hierarchical and continuous data, extending a unidimensional model to a multidimensional one [7]. The basic idea of the model [8] is that: assuming the full score of an item is *f*_*j*_, then the number of scores for item *j* is *f*_*j*_ + 1, that is 0,1,2…,*f*_*j*_. If *P*_ajt_* is the probability that the score of item *j* is greater than *t* when the ability value is *θ*_*a*_, then *P*_*aj*0_* = 1, *P*_*aj, f*__*j*__+1_* = 0. If *P*_*ajt*_ is also the probability that the score of item *j* is *t* [9], then *P*_*ajt*_ *= P*_ajt_*-*P*_*aj, t+*1_* (*t* = 0,1,2, …, *f*_*j*_), where *P*_ajt_* = 1/{1 + exp[−*Da*_*j*_(*θ*_*a*_-*b*_*jt*_)]}, in which D = 1.7, *a*_*j*_ is the discriminant degree of item *j*, *b*_*jt*_ is the difficulty when the score of item *j* is t, and the difficulty level of item *j* is monotonically increasing; that is, *b*_*j1*_ < *b*_*j2*_ < … < *b*_*j*_,_*fj*_. *P*_ajt_* corresponding to an ICC is called the Project type characteristic function in the Graded Response model.

Five parameters can be estimated in our study, namely *a*,*b*_1_,*b*_2_,*b*_3_,*b*_4_, where *b*_1_ is the parameter of difficulty level between answer 1 and answer 2, and so on, and *b*_1<_*b*_2<_*b*_3<_*b*_4_. Here *a* must be > 0.60, and b ranges from −3 to 3.

Items supported by at least five methods were retained in the final LC-PROM.

### Step 3 validation of the scale

#### Second Sampling Survey

Six hundred twenty subjects were selected in the second survey, of which 120 were controls. Inclusion and exclusion criteria did not change, nor did the survey process.

### Reliability analysis

Reliability reflects the stability and consistency of a scale. In our study, Cronbach’s α coefficients for the total scale and for each domain were calculated, to evaluate the average consistency of the items. The higher the value is, the better the reliability, but if α is too high, it suggests that the items are not simply related but overlap considerably. In the extreme case where α = 1,we should consider whether some items are redundant and could be eliminated. Here we chose 0.80 as the critical value; i.t., the measured results can be considered stable when α exceeds 0.80.

### Validity analysis

Validity, also called accuracy, is the other arm of validation of a scale, and reflects the extent to which a scale measures what it sets out to measure. Validity includes subtypes of content validity, criterion validity, construct validity, and discriminant validity. In this article, we chose to measure the latter two.

### Construct validity

This index shows whether the scale constructs match those in the initial framework. A scale with good construct validity is able to target true potential traits for measurement. Factor analysis is a major method for construct validity analysis and includes Exploratory Factor Analysis (EFA) and Confirmatory Factor Analysis (CFA). When an item collection is not based on theoretical guidance, EFA has the ability to explore the fields and dimensions belonging to a scale. However, before this study, we had reviewed the literature to formulate a scale framework, and EFA had been applied during the process of item selection, so at this stage CFA was suitable. Factor loading for every item and fit index for every domain were calculated.

### Discriminant validity

This is an index of a scale’s ability to discriminate populations with different traits through comparing test results of selected subjects. The statistical method was a simple two-independent samples *t*-test. The total scores on the LC-PROM and on each domain were compared between cases and controls to judge whether the LC-PROM could distinguish these two groups. In addition, we stratified the time that patients had been sick as less than 1 year, 1 to 3 years, 3 to 5 years, and more than 5 years. ANOVA was then applied to infer the relationship between disease course and scale score. The scale we developed had a good discriminant validity when p ≤ 0.05.

### Feasibility analysis

When a scale can be understood and completed by subjects easily, the scale is said to have strong feasibility. This property is assessed with reference to acceptance ratio, response rate, and completion time.

### Statistical software

The data analysis was conducted by SPSS16.0, Multilog7.03 and LISREL8.70.

**Fig. 1:**
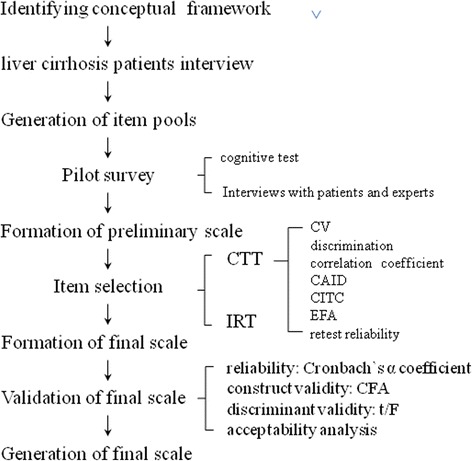
Study flow diagram

The entire study flow diagram is presented in Fig. 1.

## Results

### Generation of item pool

#### Literature review and patient interviews

Database searches revealed some liver disease-specific scales, such as the Hepatitis Quality Of Life Questionnaire (HQLQ) [10, 11], the Liver Disease Quality Of Life (LDQOL) [10, 11], the Chronic Liver Disease Questionnaire (CLDQ) [10–13], and several related questionnaires such as the WHOQOL-BREF [11], the SF-36 [10, 11], the SCL-90 [12, 13] and the Hospital Anxiety and Depression Scale (HADS) [12, 13].

The LC-PROM focused on 4 domains: Physical (PHD), Psychological (PSD), Social (SOD), and Therapeutic (TRD). This idea is based on the definition of PRO and all the specific scales for liver disease. Meanwhile, taking the Social Avoidance and Distress Scale (SAD) and the Beck Hopelessness Scale (BHS) into consideration, the LC-PROM was divided into a further 13 dimensions, and the initial item pool included 72 items (see Appendix 1). The instrument’s conceptual framework is shown in Table 1.

**Table 1:** Preconceived conceptual framework for the LC-PROM

Field	Dimension
Physical Domain(PSD)	Abdominal Symptoms (ABS)
	Skin Symptoms (SKS)
	Appetite Symptoms (APS)
	Cognitive Ability (COG)
	Independence (IND)
Psychological Domain (PSD)	Anxiety and Depression (AND)
	Confidence (CON)
	Disease Outcomes (DIO)
Social Domain (SOD)	Social Supports (SOS)
	Social Adaptation (SOA)
Therapeutic Domain (TRD)	Satisfaction (SAT)
	Compliance (COM)
	Drug Side Effects (DSE)

### Cognitive debriefing and expert discussion

The LC-PROM was regarded as clear and concise, easy to understand and easy for the patients in the cognitive debriefing to complete. Completion time was 10 min on average. Considering patients’ suggestions, we made some modifications to the instrument. Six items in PHD that described atypical symptoms and overlapped with each other were deleted. Symptoms in deleted items included, for example, oliguria, dry eyes, pale skin and mucosa, among others. We also replaced the words “hepatic region” with “right upper abdomen,” to make this text easier to understand. Similarly, two items were reduced in PSD, one item was reduced in TRD, and one item was added in SOD.

Experts agreed that the LC-PROM was reasonable in its construction framework and item attributions, and that it was comprehensive in its content. However, because this was a self-rating scale, it was determined that the items should be expressed in the first person, so a full revision was made by research group accordingly. This second draft of the preliminary LC-PROM included 64 items, 13 dimensions and four fields (see Appendix 2).

### Item reduction

#### Participant characteristics

We sampled 200 participants in this survey; 189 responded, for an acceptance rate of 94.50 %. There were 179 subjects, including 132 patients and 47 controls, whose data were available, for a final response rate of 94.71 %. Baseline data of participants are shown in Table 2. The average length of time since liver cirrhosis diagnosis was approximately 3.02 years.

**Table 2:** Baseline data for participants in pilot survey

Variables		Liver Cirrhosis	Health Control	*t/χ* ^*2*^	*P*
Age (years)		55.46 ± 11.60	51.94 ± 15.02	1.462	0.149
Gender				1.149	0.284
Male		82	25		
Female		50	22		
Height (cm)		167.46 ± 7.68	166.04 ± 7.53	1.091	0.277
Weight (kg)		62.83 ± 10.42	65.83 ± 12.79	−1.596	0.112
Drinking	never	42	25/6/12/4	9.243	0.026
	quit	28	6		
	occasional	32	12		
	always	30	4		
Smoking	never	46	26	11.299	0.023
	quit	26	10		
	<10branches/d	18	3		
	10branches/d~	14	6		
	20branches/d~	28	2		

### Item selection based on CTT and IRT

When CAID was used, we calculated the initial Cronbach’s α coefficient when all 64 items were retained; this did not result in deletion of any items, the detailed result was not shown here.

**Fig. 2:**
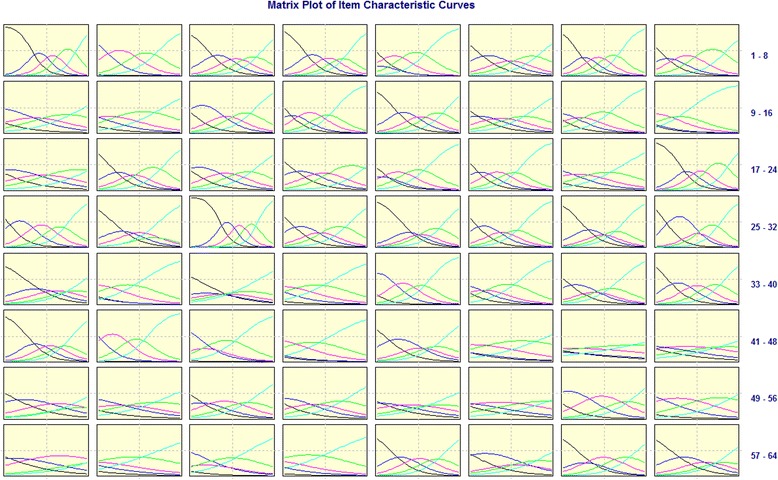
Matrix plot of item characteristic curve

In IRT a number of items were suggested for deletion: fourteen in PHD, four in PSD, and seven in TRD; and only one item was retained in SOD according to parameters *a* and *b*. Fig. 2 shows the ICC matrix.

Fifteen items were to be deleted based on statistical results, but considering the value of disease-specific symptom information and the contributions of certain items to each dimension, six items were maintained in the final version of the LC-PROM. The final version comprised 55 items within 13 dimensions belonging to 4 domains (see Appendix 2). The detailed screening process is presented in Table 3, and the final construction frame can be seen in Table 4.

**Table 3:** Item selection outcome based on CTT and IRT

Dimension	Item	SD	*t*	Factor Loading	CAID	CITC	CC	Retest Reliability	IRT					Outcome
									*a*	*b*_*1*_	*b*_*2*_	*b*_*3*_	*b*_*4*_	
ABS	PHD1	1.18	0.001	0.619	0.736	0.579	0.713	0.858	1.83	−1.07	−0.02	0.91	2.20	√
	PHD2	**0.90**	0.001	**0.474** ^**a**^	0.774	**0.348**	**0.484**	0.860	1.06	**−6.98**	−2.53	−0.43	1.41	**×**
	PHD3	1.21	0.001	**0.540** ^**a**^	0.750	0.509	0.659	0.734	1.17	−1.80	−0.34	0.86	2.20	√
	PHD4	1.25	0.001	0.671	0.756	0.478	0.639	0.802	1.37	−1.60	−0.31	0.76	1.79	√
	PHD5	**0.97**	0.001	0.697	0.752	0.505	0.628	0.817	1.15	**−3.16**	−2.50	−1.06	0.70	√
	PHD6	1.21	0.001	**0.510** ^**a**^	0.771	**0.392**	**0.566**	**0.644**	0.91	−2.58	−0.74	0.58	2.38	**×**
	PHD7	1.20	0.001	0.620	0.747	0.525	0.670	0.886	1.47	−1.99	−0.97	0.07	1.24	√
	PHD8	1.05	0.001	0.633	0.749	0.517	0.647	0.840	1.09	−2.81	−1.51	0.04	2.17	√
SKS	PHD9	1.13	0.001	0.673	0.529	**0.382**	0.706	0.779	**0.59**	**−5.48**	−1.91	0.18	2.77	√
	PHD10	1.01	0.001	0.668	0.473	0.421	0.644	0.751	0.61	**−6.19**	**−3.45**	−1.21	1.76	√
	PHD11	1.12	0.001	**0.543** ^**a**^	0.483	0.411	**0.451**	**0.649**	1.07	**−3.40**	−1.09	0.18	1.74	**×**
APS	PHD12	1.09	0.001	**0.603** ^**a**^	0.519	0.535	0.718	0.732	1.21	**−3.04**	−1.85	−0.43	0.77	√
	PHD13	1.21	0.001	**0.655** ^**a**^	0.612	**0.307**	**0.574**	0.719	1.20	−1.81	−0.36	0.75	2.16	**×**
	PHD14	1.23	0.001	0.642	0.618	**0.295**	**0.567**	0.713	0.75	**−3.40**	−1.55	0.06	1.89	**×**
	PHD15	1.03	0.001	0.672	0.584	**0.376**	**0.589**	0.767	0.92	**−4.20**	−2.45	−1.09	0.82	**×**
	PHD16	**0.89**	**0.915**	**0.528** ^**a**^	0.590	**0.367**	**0.555**	0.809	0.80	**−5.28**	**−4.08**	−2.12	−0.43	**×**
	PHD17	1.14	0.001	0.698	0.600	0.400	**0.576**	0.781	**0.57**	**−4.35**	−1.35	0.81	**3.86**	√
COG	PHD18	1.20	0.001	0.816	0.438	0.567	0.831	**0.692**	1.15	−2.22	−0.93	0.13	1.82	√
	PHD19	1.14	0.001	0.848	0.419	0.585	0.829	0.727	0.87	**−3.59**	−1.33	0.28	2.03	√
	PHD20	1.12	0.001	**0.584** ^**a**^	**0.779**	**0.302**	0.657	0.840	0.87	−2.74	−0.94	0.68	**3.15**	**×**
IND	PHD21	1.07	0.033	0.633	0.635	0.525	0.788	0.815	1.05	**−3.25**	−2.23	−0.80	0.81	√
	PHD22	1.14	0.002	0.708	0.582	0.566	0.824	0.813	1.18	−2.92	−1.67	−0.40	0.82	√
	PHD23	1.07	0.018	0.777	0.653	0.509	0.780	0.820	0.64	**−5.93**	−2.96	−0.93	1.40	√
AND	PSD1	1.19	0.001	**0.504** ^**a**^	0.807	0.532	0.645	0.774	1.54	−1.26	−0.26	0.77	2.33	√
	PSD2	1.07	0.001	0.704	0.805	0.558	0.656	0.768	1.33	−2.85	−1.11	0.33	1.62	√
	PSD3	1.39	0.001	0.606	0.814	0.481	0.622	**0.628**	0.97	−1.96	−0.61	0.61	1.41	√
	PSD4	1.21	0.001	0.680	0.797	0.624	0.721	0.829	2.16	−0.91	0.09	0.95	1.86	√
	PSD5	1.19	0.001	0.611	0.809	0.517	0.633	0.814	1.04	−2.64	−0.97	0.17	1.85	√
	PSD6	1.36	0.001	0.699	0.804	0.555	0.679	0.781	1.19	−1.24	−0.23	0.64	1.96	√
	PSD7	1.20	0.001	0.658	0.809	0.513	0.631	0.817	1.17	−2.78	−1.24	−0.20	0.99	√
	PSD8	1.36	0.001	0.589	0.809	0.516	0.648	**0.626**	1.17	−1.74	−0.51	0.34	1.44	√
	PSD9	1.16	0.001	**0.597** ^**a**^	0.813	0.476	**0.596**	0.858	1.45	−2.31	−0.34	0.42	1.74	√
CON	PSD10	1.34	0.001	0.691	**0.624**	**0.237**	**0.511**	0.830	0.78	−1.59	0.05	1.45	2.85	√
	PSD11	**0.94**	0.001	**0.623** ^**a**^	0.590	**0.328**	**0.509**	0.844	0.68	**−5.55**	**−4.47**	−2.09	0.33	**×**
	PSD12	1.43	0.001	0.650	0.611	**0.283**	**0.564**	0.722	0.60	−2.71	1.20	0.21	1.95	√
	PSD13	**0.93**	0.001	0.676	0.591	**0.326**	**0.505**	0.860	0.61	**−7.67**	**−4.43**	−1.99	0.69	**×**
	PSD14	1.02	0.001	**0.632**	0.546	0.471	0.640	0.804	1.24	**−4.19**	−1.87	−0.43	0.85	√
	PSD15	1.03	0.001	0.634	0.551	0.454	0.629	0.776	0.85	**−4.51**	−2.66	−1.29	0.70	√
	PSD16	1.25	0.001	**0.504** ^**a**^	0.587	**0.334**	**0.571**	0.721	0.95	−2.97	−1.23	−0.20	1.19	**×**
DIO	PSD17	1.16	0.001	0.749	-	0.461	0.836	0.736	1.20	−2.17	−0.65	0.66	2.05	√
	PSD18	1.31	0.001	0.686	-	0.461	0.873	0.782	1.25	−1.40	−0.23	0.82	1.85	√
SOS	SOD1	**0.85**	**0.836**	0.719	0.476	0.480	0.702	0.854	1.23	**−6.39**	−2.98	−1.00	0.55	**×**
	SOD2	**0.95**	**0.492**	**0.606** ^**a**^	0.567	**0.336**	0.628	0.842	0.89	**−7.79**	−2.62	−1.43	0.58	**×**
	SOD3	**0.90**	**0.480**	0.767	0.462	0.489	0.720	0.855	0.60	**−6.92**	**−5.35**	−2.33	0.36	**×**
	SOD4	1.26	0.036	**0.579** ^**a**^	**0.636**	**0.296**	0.690	0.785	0.94	−2.47	−0.44	0.73	2.05	√
SOA	SOD5	1.06	0.001	0.786	0.797	0.543	0.646	0.731	**0.44**	**−6.55**	**−4.41**	−1.26	2.72	√
	SOD6	1.27	0.001	0.835	0.792	0.576	0.692	0.741	**0.25**	**−8.24**	**−4.67**	−0.42	**4.89**	√
	SOD7	1.22	0.001	**0.438** ^**a**^	0.804	0.481	0.611	0.771	**0.38**	**−5.95**	−2.52	0.75	**3.99**	√
	SOD8	1.26	0.001	**0.385**	0.803	0.488	0.621	0.849	**0.57**	**−3.06**	−0.25	1.91	**3.49**	√
	SOD9	1.26	0.001	0.821	0.809	0.433	**0.566**	0.742	**0.46**	**−6.51**	−2.60	−0.23	2.69	√
	SOD10	1.19	0.001	0.630	0.793	0.575	0.685	**0.638**	0.79	**−3.19**	−1.53	0.32	2.02	√
	SOD11	1.18	0.017	0.693	0.803	0.490	0.614	0.757	0.67	**−4.33**	−1.78	−0.01	2.18	√
	SOD12	1.28	0.015	0.748	0.799	0.525	0.653	0.915	**0.49**	**−4.45**	−1.79	0.52	2.82	√
	SOD13	1.17	0.001	0.713	0.796	0.545	0.659	0.832	**0.41**	**−5.81**	−2.58	0.77	**4.31**	√
SAT	TRD1	1.03	0.001	0.755	0.505	0.434	0.752	0.739	0.88	**−4.05**	−1.27	0.92	2.53	√
	TRD2	**0.98**	0.009	0.722	0.498	0.441	0.742	0.759	**0.58**	**−7.28**	−2.96	0.05	3.16	√
	TRD3	1.13	0.001	0.720	0.552	0.406	0.765	0.878	**0.46**	**−4.13**	−0.88	2.86	**5.24**	√
COM	TRD4	1.03	**0.537**	0.794	0.524	0.449	0.767	0.825	**0.42**	**−8.59**	**−5.10**	−2.36	1.43	√
	TRD5	1.11	0.039	0.617	0.587	0.413	0.771	0.728	0.61	**−6.83**	−2.68	−1.25	1.19	√
	TRD6	**0.89**	0.043	0.778	0.499	0.479	0.747	0.834	**0.49**	**−9.49**	**−5.76**	**−3.07**	0.59	√
DSE	TRD7	1.23	0.001	0.801	0.444	0.494	0.744	0.716	1.06	−2.04	−0.57	0.80	2.10	√
	TRD8	1.26	0.001	0.683	0.504	0.417	0.701	**0.632**	0.61	**−3.48**	−0.34	1.12	**3.19**	√
	TRD9	1.23	0.005	**0.487** ^**a**^	0.564	**0.338**	0.640	**0.669**	0.95	−2.00	−0.86	0.77	2.42	**×**
	TRD10	1.28	0.006	0.631	0.602	**0.290**	0.618	0.702	0.82	−1.93	−0.03	1.32	2.66	√

Note: “CC” is short of correlation coefficient, boldface means items which suggested to delete by certain method, “^a^” means items that measure cross dimensions, “√”means maintain, “×”means delete

**Table 4:** Construction frame of the final LC-PROM

Domain	Dimension	Item
Physical Domain (PHD)	Abdominal Symptoms (ABS)	1-,2-,3-,4-,5-,6-
	Skin Symptom (SKS)	7-,8-
	Appetite Symptoms (APS)	9-,10-,11-,12-,13-
	Cognitive Ability (COG)	14-,15-
	Independence (IND)	16+,17+,18+
Psycological Domain (PSD)	Anxiety and Depression (AND)	1-,2-,3-,4-,5-,6-,7-,8-,9-
	Confidence (CON)	10-,11-,12-,13-,14-
	Disease Outcomes (DIO)	15-,16-
Social Domain (SOD)	Social Supports (SOS)	1+,2+,3+
	Social Adaptation (SOA)	4-,5-,6-,7-,8-,9-,10-,11+,12-
Therapeutic Domain (TRD)	Satisfaction (SAT)	1+,2+,3+
	Compliance (COM)	4+,5+,6+
	Drug Side Effects (DSE)	7-,8-,9-

Note: “+”means positive item, “-”means negative item

### Validation of LC-PROM

#### Demographic characteristics

Another 620 subjects (500 cases and 120 controls) were sampled for the validation. Of the 598 who responded, 576 produced valid data for analysis (464 cases and 112 controls). Participant characteristics are presented in Table 5.

**Table 5:** Demographic characteristics of 464 patients and 112 controls in LC-PROM validation

Variables		Liver Cirrhosis	Health Control	_*t/χ*_^*2*^	*P*
Age (Years)		55.62 ± 11.06	53.81 ± 11.38	1.547	0.123
Gender	Male	283	64	0.558	0.455
	Female	181	48		
Height (cm)		166.96 ± 7.76	167.73 ± 6.98	−0.967	0.334
Weight (kg)		62.08 ± 10.34	66.66 ± 9.95	−4.241	0.001
Drinking	Never	146	62	29.968	0.001
	Quit	104	10		
	Occasional	127	32		
	Always	87	8		
Smoking	Never	186	51	23.232	0.001
	Quit	162	21		
	<10branches/d	48	7		
	10branches/d~	32	20		
	20branches/d~	36	13		

As Table 5 shows, males were more numerous than females; subjects’ average age was 50–55 years. There were no statistically significant differences in the distributions of gender, age, or height between the two groups. LC patients had a higher proportion of smoking and drinking, and lower weight. These characteristics are consistent with risk factors for LC. Among the subjects, 269 patients had been sick for 1 to 5 years, the number of patients who suffered from LC less than 1 year and more than 5 years were 97 and 98 respectively, the average length of time was 3.70 years.

### Reliability analysis

Cronbach’s α coefficient is one of the indicators for evaluating reliability, with a generally acceptable value of greater than 0.70. Our LC-PROM met this standard, except in the TRD domain (see Table 6).

**Table 6:** Cronbach’s α coefficient of four domains and total scale

Domain	The number of item	Cronbach’s α coefficient
Physical	18	0.923
Psychological	16	0.930
Social	12	0.840
Therapeutic	9	0.698
Total	55	0.952

### Validity analysis

**a.** Construct validity: Results of CFA are listed in Tables 7 and 8, and show factor loadings of items and goodness of fit of domains in the final LC-PROM.

**Table 7:** Maximum Likelihood Estimation of CFA for LC-PROM

Domain	Dimension	Item	Standard Factor Loading	Nonstandard Factor Loading	SE	*t*
PHD	ABS	PHD1	0.81	0.95	0.04	22.79
		PHD2	0.86	0.99	0.04	25.34
		PHD3	0.83	0.99	0.04	23.77
		PHD4	0.75	0.63	0.03	20.54
		PHD5	0.63	0.61	0.04	16.19
		PHD6	0.74	0.79	0.04	20.18
	SKS	PHD7	0.91	0.97	0.04	23.98
		PHD8	0.76	0.78	0.04	19.59
	APS	PHD9	0.51	0.50	0.04	12.06
		PHD10	0.63	0.63	0.04	15.66
		PHD11	0.69	0.77	0.04	17.52
		PHD12	0.71	0.72	0.04	18.17
		PHD13	0.65	0.65	0.04	16.17
	COG	PHD14	0.89	0.89	0.03	25.82
		PHD15	0.93	0.91	0.03	27.43
	IND	PHD16	0.76	0.69	0.04	19.38
		PHD17	0.61	0.60	0.04	14.70
		PHD18	0.82	0.75	0.04	21.13
PSD	AND	PSD1	0.83	0.95	0.04	23.99
		PSD2	0.65	0.69	0.04	17.07
		PSD3	0.89	1.04	0.04	27.23
		PSD4	0.80	0.91	0.04	22.71
		PSD5	0.69	0.72	0.04	18.65
		PSD6	0.76	0.87	0.04	21.22
		PSD7	0.66	0.72	0.04	17.59
		PSD8	0.67	0.72	0.04	17.92
		PSD9	0.83	0.95	0.04	23.91
	CON	PSD10	0.80	0.93	0.04	22.46
		PSD11	0.87	0.95	0.04	25.62
		PSD12	0.61	0.52	0.03	15.39
		PSD13	0.59	0.51	0.03	14.82
		PSD14	0.86	0.99	0.04	24.79
	DIO	PSD15	0.80	0.95	0.05	19.76
		PSD16	0.83	0.99	0.05	20.54
SOD	SOS	SOD1	0.62	0.51	0.06	8.42
		SOD2	0.59	0.49	0.06	8.31
		SOD3	0.35	0.37	0.06	6.26
	SOA	SOD4	0.57	0.59	0.04	14.39
		SOD5	0.86	0.96	0.04	24.93
		SOD6	0.66	0.77	0.05	17.14
		SOD7	0.70	0.91	0.05	18.57
		SOD8	0.52	0.51	0.04	12.67
		SOD9	0.72	0.80	0.04	19.26
		SOD10	0.72	0.73	0.04	19.40
		SOD11	0.67	0.72	0.04	17.38
		SOD12	0.64	0.73	0.04	16.40
TRD	SAT	TRD1	0.83	0.70	0.04	19.34
		TRD2	0.66	0.59	0.04	15.54
		TRD3	0.66	0.69	0.04	15.44
	COM	TRD4	0.59	0.50	0.04	11.34
		TRD5	0.69	0.69	0.06	12.43
		TRD6	0.56	0.42	0.04	11.00
	DSE	TRD7	0.79	0.87	0.05	18.92
		TRD8	0.80	0.86	0.04	19.56
		TRD9	0.63	0.70	0.05	14.99

**Table 8:** Goodness of fit statistics of LC-PROM

	*RMSEA*	*RMR*	*NFI*	*NNFI*	*CFI*	*IFI*
PHD	0.06	0.05	0.97	0.98	0.98	0.98
PSD	0.07	0.06	0.97	0.98	0.98	0.98
SOD	0.09	0.05	0.95	0.95	0.96	0.96
TRD	0.02	0.03	0.98	0.99	1.00	1.00
Total	0.06	0.06	0.91	0.93	0.94	0.94

As the tables show, standard factor loadings of each item were above 0.50, except for SOD3; therefore, the goodness of fit for LC-PROM is satisfactory.

**b.** Discriminant validity: Discriminant validity analysis was conducted by comparing average scores across different domains as well as total scale scores between patients with various disease courses and the health controls.

In Table 9, the scores of patients are lower than those of controls, suggesting that LC severely affected patients’ quality of life. With SOD as the exception, scores were significantly different, as seen in Table 10, and longer clinical courses were associated with lower scores. Perhaps because LC is the final stage of liver disease progression, by the time patients have received a definite diagnosis, they may already have lost the ability to engage in social activity; therefore scores in this domain did not differ. Of course, measurement error cannot be excluded as an explanation, but it had little effect on discriminant validity. In summary, the LC-PROM was well able to differentiate health and LC patients in varying clinical courses.

**Table 9:** Score comparisons between LC patients and health controls

	Liver Cirrhosis (n = 464)	Health Controls (n = 112)	*t*	*P*
Physical	63.68 ± 11.12	80.74 ± 3.11	28.73	<0.001
Psychological	52.26 ± 10.00	73.29 ± 2.76	39.52	<0.001
Social	42.29 ± 7.47	50.78 ± 3.81	17.01	<0.001
Therapeutic	30.77 ± 4.53	36.19 ± 2.61	16.73	<0.001
Total	189.00.24 ± 24.79	241.01 ± 6.76	39.51	<0.001

**Table 10:** Scores obtained using the LC-PROM instrument in varying disease courses of LC

	<1 year	1 year~	3 ~ years	5 ~ years	*F*	*P*
	(n = 97)	(n = 133)	(n = 136)	(n = 98)		
Physical	67.62 ± 9.70	66.23 ± 10.65	62.15 ± 10.25	58.46 ± 11.87	15.83	<0.001
Psychological	53.82 ± 9.38	53.29 ± 10.59	51.98 ± 9.17	49.70 ± 10.49	3.49	0.016
Social	42.40 ± 6.52	43.41 ± 7.69	41.22 ± 7.42	42.13 ± 7.99	1.95	0.120
Therapeutic	32.07 ± 3.75	31.20 ± 4.81	30.32 ± 4.25	29.50 ± 4.83	6.32	<0.001
Total	195.92 ± 21.53	194.13 ± 25.10	185.67 ± 21.69	179.80 ± 27.91	10.32	<0.001

### Feasibility analysis

The acceptance rate and response rate for the LC-PROM tool were 96.45 % and 92.32 %, respectively. Its average completion time was 10 min.

## Discussion

LC is a chronic disease characterized by progressive liver injury which imposes a heavy burden on medical and health services. Bajaj J. S. etal revealed that patients had significant impairment on all domains apart from anger and anxiety compared with caregivers and US norms. Decompensated patients had significantly worse sleep, pain, social and physical function scores compared with compensated ones [14]. Therefore, objective evaluation of clinical effects and patients health conditions is critically important.

We performed reviews of the literature, then collected symptoms of greatest concern and with greatest likelihood of improvement, along with psychological conditions and life states from the patients’ perspective. From these, we formed the preliminary item pool for the LC-PROM instrument. Cognitive debriefing and discussions with experts were employed to ensure reasonableness of the conceptual and the structural framework. Next we applied this scale to two samples (n_1_ = 120, n_2_ = 620) that represented different populations. We considered seven statistical methods and clinical relevance when selecting final items for this tool. In current study, the final version of the LC-PROM comprised 55 items in 4 domains (18 items in PHD, 16 items in PSD, 12 items in SOD, 9 items in TRD) that represent 13 dimensions. Validation of reliability, validity, and feasibility indicated that the LC-PROM was accurate, reliable and easy to use, showing great potential for clinical application.

Through our literature search, we confirmed that the LC-PROM instrument is the first specific scale for LC. The existing PROs for liver diseases are adapted from quality of life measurement scales that are classified as a universal QOL scale and a specific HRQOL scale. For example, WHO Quality of Life-BREF(WHOQOL-BREF), Short Form 36 (SF-36), Nottingham Health Profile (NHP),and the sickness impact profile(SIP) are universal scales, and the Chronic Liver Disease Questionnaire (CLDQ), Hepatitis Quality Of Life Questionnaire (HQLQ), and Liver Disease Quality Of Life (LDQOL) are specific HRQOL scales. All the scales mentioned above have different degrees of defects and in any case do not apply to LC patients. Some studies have indicated that the WHOQOL is widely used by researchers to study QOL of liver transplant recipients, while the NHP focuses on more severe levels of disability and has thus has been known to be less sensitive to changes in conditions where effects are relatively mild [15, 16]. The SIP, in contrast, has a broad coverage of topics, but is therefore very long [17]. The SF-36 is applicable to a broader range of conditions, but has the common disadvantage of generic instruments; namely, they are not designed to identify disease-specific domains that may be important to establish clinical changes [18]. The HQLQ consists of the widely validated generic SF-36 with five added disease-specific subscales, but it excludes patients with a chronic liver disease other than HCV. The CLDQ is a short and therefore feasible questionnaire, but is unable to discriminate between more advanced stages of liver disease. The LDQOL addresses a variety of domains, but is therefore very long (101 items) [10]. The LDSI 2.0 developed by Van der Plas etal. is short, straightforward(only 18 items) and focuses on symptom severity and symptom hindrance, evaluating how patients experience these specific symptoms during daily activities[19]. But in this study, we intend to measure other aspects in addition to symptoms. The translated CLDQ is also used to measure quality of life of Hepatitis B patients [20], and although its reliability and validity have been evaluated, the cultural gap is difficult to bridge. In addition, the instrument has some inherent defects that make it inapplicable to LC patients.

The above-mentioned instruments are designed for chronic liver disease, but not for LC specifically. There is difference between these two disease types. Another point worth noting is that Japanese-related research has found no statistically significant differences among different severity levels of liver disease [13]. However, the LC-PROM tool differs from the scale these researchers used, which was translated directly from English. The LC-PROM is designed specifically for LC, and its item pool took shape through deep interviewing and cognitive testing of patients. Therefore, our instrument may be accepted by respondents more easily, and it performs better for measuring patients’ health status.

At present, liver disease questionnaires mainly focus on “physical”, “psychological” and “limitation” dimensions. The CLDQ also includes just six subdomains: abdominal symptoms, fatigue, systemic symptoms, activity, emotional function, and worry [21]. The LC-PROM contains a vital addition—a therapeutic domain to obtain information about treatment satisfaction, compliance and drug side effects. The satisfaction with treatment is the major outcome index in new drug clinical trials; this additional field provides information about effects that the trial drug has on targeted patients’ health (such as appetite symptoms, cognitive ability, independence, anxiety and depression, and confidence) and points out the compliance characteristics of the new drug among patients. These are valuable data for clinical therapeutic drug development. Additionally, optimal therapy can be selected according to these measurement data. In the social domain, the family relationship was emphasized reminding readers of the important role of family support during patient recovery.

During the item selection process, in addition to using subjective methods like cognitive tests and expert discussions, we combined seven kinds of statistical methods to refine the item pool to ensure that items retained were maximally accurate, objective and reliable. Methods employed to develop related scales are still limited to CTT. The innovation of our study is to put IRT into use in addition to CTT. IRT is able to make up for some disadvantages of CTT, allowing acquisition of items that reflect potential traits of the population more accurately.

The instrument demonstrated excellent discriminant ability among LC patients with varying courses of disease. At a basic level, physicians can judge different stages of disease according to the results of the LC-PROM. This will save time relative to the method of full reliance on laboratory indicators.

In a word, the LC-PROM instrument we developed fills a gap in patient-reported clinical outcomes of LC, and lacks the deficiencies seen with existing liver disease PRO tools. It also has the capacity to discriminate disease course, and to evaluate clinical effects and HRQOL accurately; therefore, it will provide valuable data to new drug development for LC.

### However, this study still has quite a few limitations that will be addressed and improved in further research

To begin, Cronbach’s α coefficient for the therapeutic domain in the LC-PROM was less than 0.70, which suggests that the internal coherence of this domain needs to be improved further. As seen in the CFA results, the factor loading for item SOD3 (“I have told my worries to my family”) is only 0.35, but in consideration of its special meaning—support from family during illness—we kept it in the final scale. In fact, in the item selection phase, SOD3 was already suggested for deletion with SOD1 (“Friends and relatives take care of my disease”), but we maintained this item for the same reason. Besides, there is no items about sexual function in the scale. The participants expressed that these types of questions were a little sensitive and that it was difficult to respond. We worried about the low response rate and bad overall reliability and validity; therefore we did not include these information in the scale. In order to expand the scope of use, a scale containing this item will be generated in a revised version.

A second limitation relates to criterion validity. The LC-PROM instrument was designed for LC patients, and although participants at different stages of the clinical course were sampled, LC is the final stage of liver disease progression, and patients are often too weak to complete a lengthy scale. Introducing too many tests leads to test fatigue and noncompliance, which increases both survey cost and patients’ exhaustion levels; both influence survey results negatively. Therefore, we did not conduct criterion validity analysis in this study;

Last but not the least, because of limited resources, our samples were recruited from restricted regions and therefore may not be representative of all patients with LC.

## Conclusions

Our study provides strong evidence for excellent reliability and validity of a PRO instrument for LC. We do not suggest that the LC-PROM can replace other related questionnaires on liver disease, but it can obtain valuable information on patients’ health conditions, evaluate clinical effects, inform therapeutic method selection and new drug development, as well as health service deployment and clinical research.

## Appendix 1

**Table 11:** Formation of LC-PROM item pool of 72

Item	Item
PHD1. Did you feel abdominal distension?	PSD8. Did you feel angry easier than usual?
PHD2. Did you have diarrhea?	PSD9. Were you more indifference to things than ever?
PHD3. Did you feel fatigue often?	PSD10. Did you worry about that your liver disease would infect your family?
PHD4. Did you have ascites?	PSD11. Did you feel vague about your future?
PHD5. Did you feel bellyache often?	PSD12. Did you think other people would live better if you died?
PHD6. Did you have pain in your liver area?	PSD13. Did you lose interests in what interested you before?
PHD7. Did you have melena?	PSD14 I thought I didn’t need any treatments because I wouldn’t be cured
PHD8. Did you have constipation?	PSD15. I didn’t have any hope about my future
PHD9. Did you have oliguria or no urine?	PSD16. Did you feel unuseful about yourself
PHD10. Did you have haematemesis?	PSD17. I was pessimistic and there was nothing to make me happy
PHD11. Did you vomit?	PSD18. Were you worried about that your liver cirrhosis would cause more serious disease (like cancer)?
PHD12. Were you pale in skin and mucosa?	PSD19. Did you feel anxious about your disease outcome?
PHD13. Did your skin turn yellow?	PSD20. Did you have determination to fight with the disease?
PHD14. Did you look dark and dull?	SOD1. Did your relatives and friends care about your disease?
PHD15. Did you feel dry in your eyes?	SOD2. Did your family take care of your living actively?
PHD16. Did you feel itchy in skin?	SOD3. Did your family comfort you?
PHD17. Did you have symptoms of bleeding gums or nasal bleeding?	SOD4. Did you tell your worries to your family?
PHD18. Did you feel loss of appetite?	SOD5. Did you feel uncomfortable about friends’ strange eyes or attitude after you were ill?
PHD19. Did you have abnormal tastes (bitter, sweet, or stick)	SOD6. Did you feel someone avoided you deliberately?
PHD20. Did you feel sleepy in daytime?	SOD7. Did you avoid social occasions or activities because of disease (like parties)?
PHD21. Did you feel dizzy?	SOD8. Did you give up your hobbies before (like dancing, playing cards)
PHD22. Were you losing weight?	SOD9. Were you lack of initiative in social life?
PHD23. Did you feel breath hard?	SOD10. Did the disease affect your interpersonal relationships?
PHD24. Did you have edema in your legs?	SOD11. Did you continue to go to work at your original workplace?
PHD25. Did you feel mental decline?	SOD12. Were you worried about the disease would affect your work or promotion?
PHD26. Did you feel forgetful?	TRD1. Were you satisfied with current treatments?
PHD27. Could you do shopping as usual (like buying vegetables or daily necessities)?	TRD2. Were you satisfied with the medical services?
PHD28. Can you do simple housework (like cooking, dumping)	TRD3. Were you satisfied with the health care costs?
PHD29. Can you do some simple outdoor activities (like walking)?	TRD4. Can you follow the doctor’s advices to give up bad living habits ?
PSD1. Did you feel anxious easier than usual?	TRD5. Can you go to visit doctors regularly?
PSD2. Did you feel upset or fear easily?	TRD6. Can you take medicine following the doctor’s advice (in hospital or at home)?
PSD3. Were you difficult to fall asleep, dreaming and waking up early?	TRD7. Were you tired of taking medicine often?
PSD4. Did you feel restless and have to activate?	TRD8. Did you feel annoyed about drawing blood and taking B ultrasonic examination?
PSD5. Did you feel in a blue mood?	TRD9. Did you know side effects of pharmaceutical drugs?
PSD6. Did you feel like crying?	TRD10. Were you worried about side effects of drugs?
PSD7. Did you often feel lonely?	TRD11. Did treatments increase your life confidence?

## Appendix 2

**Table 12:** Final version of LC-PROM

A. Physical Domain					
	Never	Occasionally	About half of the time	Often	Almost everyday
1. I felt abdominal distension	0	1	2	3	4
2. I felt fatigue	0	1	2	3	4
3. I had pain in right upper quadrant	0	1	2	3	4
4. I had melena	0	1	2	3	4
5. I had haematemesis	0	1	2	3	4
6. I vomited or nauseated	0	1	2	3	4
7. My face looked dark and dull	0	1	2	3	4
8. I felt itchy in skin	0	1	2	3	4
9. I felt loss of appetite	0	1	2	3	4
10. I had abnormal tastes (bitter, sweet, or stick)	0	1	2	3	4
11. I felt sleepy in daytime	0	1	2	3	4
12. I was losing weight	0	1	2	3	4
13. I had edema in my legs	0	1	2	3	4
14. I felt forgetful	0	1	2	3	4
15. I was slow in reacting	0	1	2	3	4
	Unable	Occasionally	About half of the time	Often	Always
16. I can do shopping as usual (like buying vegetables or daily necessities)	0	1	2	3	4
17. I can do simple housework (like cooking, dumping)	0	1	2	3	4
18. I can do some simple outdoor activities (like walking)	0	1	2	3	4
B. Psychological Domain					
	Never	Occasionally	About half of the time	Often	Almost everyday
1. I felt anxious easier than usual	0	1	2	3	4
2. I felt upset or fear easily	0	1	2	3	4
3. I was annoyed at abdominal pain and indigestion	0	1	2	3	4
4. I had poor sleep at night	0	1	2	3	4
5. I felt restless and had to activate	0	1	2	3	4
6. I felt in a blue mood	0	1	2	3	4
7. I felt like crying	0	1	2	3	4
8. I felt angry easier than usual	0	1	2	3	4
9. I was worried about that my liver disease would infect my family	0	1	2	3	4
10. I felt vague about my future	0	1	2	3	4
11. I lost interests in what interested me before	0	1	2	3	4
12. I didn’t have any hope about my future	0	1	2	3	4
13. I felt unuseful about myself	0	1	2	3	4
14. I was pessimistic and there was nothing to make me happy	0	1	2	3	4
15. I was worried about that my liver cirrhosis would cause more serious disease (like cancer)	0	1	2	3	4
16. I felt anxious about my disease outcome	0	1	2	3	4
C. Social Domain					
	Never	Occasionally	About half of the time	Often	Almost everyday
1. My relatives and friends cared about my disease	0	1	2	3	4
2. My family comforted me	0	1	2	3	4
3. I told my worries to my family	0	1	2	3	4
	Never	Occasionally	About half of the time	Often	Almost everyday
4. I felt uncomfortable about friends’ strange eyes or attitude after I was ill	0	1	2	3	4
5. I felt someone avoided me deliberately	0	1	2	3	4
6. I avoided social occasions or activities because of disease (like parties)	0	1	2	3	4
7. I gave up my hobbies before (like dancing, playing chess)	0	1	2	3	4
8. I was lack of initiative in social life	0	1	2	3	4
9. The disease affected my interpersonal relationships	0	1	2	3	4
10. It was easy to relax being with others	0	1	2	3	4
11. I continued to go to work at my original workplace	0	1	2	3	4
12. I was worried about that the disease would affect my work, promotion or work force	0	1	2	3	4
D. Therapeutic Domain					
	Very dissatisfied	Dissatisfied	General	Satisfied	Very satisfied
1. I was satisfied with current treatments	0	1	2	3	4
2. I was satisfied with the medical services	0	1	2	3	4
3. I was satisfied with the health care costs	0	1	2	3	4
	Unable	Occasionally	About half of the time	Often	Always
4. I can follow the doctor’s advice to give up bad living habits	0	1	2	3	4
5. I can go to visit doctors regularly	0	1	2	3	4
6. I can take medicine following the doctor’s advice (in hospital or at home)	0	1	2	3	4
	Never	Occasionally	About half of the time	Often	Almost everyday
7. I was tired of taking medicine often	0	1	2	3	4
8. I felt annoyed about drawing blood and taking B ultrasonic examination	0	1	2	3	4
9. I was worried about side effects of drugs	0	1	2	3	4

## Abbreviations

BHS: beck hopelessness scale
CAID: cronbach’s α if item deleted
CFA: confirmatory factor analysis
CFI: comparative fit index
CITC: corrected item-total correlation
CLDQ: the chronic liver disease questionnaire
CTT: classical test theory
EFA: exploratory factor analysis
ERIQA: European regulatory issues on quality of life assessment group
FDA: food and drug administration
GT: generalizability theory
HADS: the hospital anxiety and depression scale
HQLQ: hepatitis quality of life questionnaire
HRQoL: health-related quality of life
ICC: item characteristic curve
IFI: incremental fit index
IRT: item response theory
ISPOR: international society for pharmacoeconomics and outcomes research
ISQOL: international society for quality of life studies
LC: liver cirrhosis
LC-PROM: liver cirrhosis patient-reported outcome measure
LDQOL: liver disease quality of life
NFI: normed fit index
NHP: Nottingham health profile
NNFI: non-normed fit index
PHD: physical domain
PRO: patient-reported outcome
PSD: psychological domain
QOL: quality of life
RMR: root mean square residual
RMSEA: root mean square error of approximation
SAD: social avoidance and distress scale
SCL-90: symptom check list-90
SF-36: short form 36
SIP: the sickness impact profile
SOD: social domain
TRD: therapeutic domain
WHOQOL-BREF: WHO quality of life-bref
